# Reduced activation in isometric muscle action after lengthening contractions is not accompanied by reduced performance fatigability

**DOI:** 10.1038/srep39052

**Published:** 2016-12-14

**Authors:** W. Seiberl, D. Hahn, F. K. Paternoster

**Affiliations:** 1 Biomechanics in Sports, Technical University of Munich, Germany; 2 Human Movement Science, Ruhr-University Bochum, Germany; 3 University of Queensland, School of Human Movement Studies and Nutrition Sciences, Brisbane, Australia

## Abstract

After active lengthening contractions, a given amount of force can be maintained with less muscle activation compared to pure isometric contractions at the same muscle length and intensity. This increase in neuromuscular efficiency is associated with mechanisms of stretch-induced residual force enhancement. We hypothesized that stretch-related increase in neuromuscular efficiency reduces fatigability of a muscle during submaximal contractions. 13 subjects performed 60 s isometric knee extensions at 60% of maximum voluntary contraction (MVC) with and without prior stretch (60°/s, 20°). Each 60 s trial was preceded and followed by neuromuscular tests consisting of MVCs, voluntary activation (VA) and resting twitches (RT), and there was 4 h rest between sets. We found a significant (p = 0.036) 10% reduction of quadriceps net-EMG after lengthening compared to pure isometric trials. However, increase in neuromuscular efficiency did not influence the development of fatigue. Albeit we found severe reduction of MVC (30%), RT (30%) and VA (5%) after fatiguing trials, there were no differences between conditions with and without lengthening. As the number of subjects showing no activation reduction increased with increasing contraction time, intensity may have been too strenuous in both types of contractions, such that a distinction between different states of fatigue was not possible anymore.

When an active muscle is stretched the resulting post-eccentric steady-state force is greater than an isometric force at corresponding muscle length and activation^1^,^2^. Numerous observations on all structural levels of muscle confirm specific characteristics of this phenomenon, referred to in the literature as residual force enhancement (RFE). Most findings show RFE to increase with increasing lengthening amplitudes^1^,^3^,^4^,^5^,^6^ and to be independent of velocity of stretch^5^,^7^. RFE occurs at all muscle lengths^1^,^8^,^9^, lasts as long as the muscle is kept active (long lasting) and is instantaneously eliminated by deactivation of the muscle^3^,^10^.

Research working with animal models and isolated fiber preparations in combination with histological examinations focuses on decoding the mechanisms generating RFE^11^,^12^,^13^,^14^. Underlying mechanisms are still not fully understood and a combination of active and passive components are discussed in literature, including half sarcomere non-uniformities, increase in the number of attached cross-bridges or in the average cross-bridge force, and CA^2+^-dependent titin stiffness modulation^2^,^15^,^16^,^17^.

Besides the identification of RFE mechanisms, the role that RFE plays in natural *in vivo* muscle function is subject of investigation. There is broad evidence that RFE is present in stimulated as well as voluntary muscle action of small and large human muscles, as well as in multi-joint leg extensions^18^,^19^,^20^,^21^,^22^,^23^,^24^,^25^,^26^. From an evolutionary point of view, it is questionable if benefits concerning RFE lay in the increase of the maximum force capacity of an isomeric contraction following eccentric lengthening. Recent studies^27^,^28^ confirm early proposed but barely addressed ideas that mechanisms underlying RFE contribute to performance enhancement associated with muscles undergoing stretch-shortening cycles^29^. Furthermore, it is well documented that on a submaximal level, for a given amount of force, less muscle activation is required after active lengthening for maintaining a given force output^30^,^31^,^32^,^33^. The characteristics of this stretch-induced activation reduction (AR) were associated with increased neuromuscular efficiency and reduced metabolic costs^30^,^32^,^34^, presumably optimizing the economy of muscle function^35^. If so, it may be reasoned that AR is beneficial for the resistance to neuromuscular fatigue during or after prolonged muscle action that is preceded by an eccentric lengthening contraction.

Muscle fatigue is described as an exercise-induced limitation of performance and measurable as a reduction in the ability of a muscle or muscle group to maintain a certain force over time, or a change in the myoelectric pattern of muscle activation^36^,^37^,^38^. The causes of muscle fatigue may be manifold, but primary mechanisms are associated with disturbances of the central nervous system^36^ and/or impairments of the contractile machinery within the muscle^39^,^40^, referred to as central and peripheral fatigue, respectively. Just recently, terms of performance fatigability and perceived fatigability were suggested to better describe the broad phenomenology of fatigue during human performance^41^,^42^. According to this, performance fatigability depends on the contractile and nervous capabilities to maintain a task against factors modulating the development of fatigue, such as calcium kinetics or activation patterns^41^. Concerning voluntary muscle activation, an influence of RFE-mechanisms on factors modulating performance fatigability was shown in several *in vivo* studies^30^,^32^,^43^.

The findings that active lengthening of a muscle leads to stretch-induced optimization of neuromuscular efficiency^18^,^20^,^30^,^31^,^32^,^33^,^44^ and reduced ATPase activity per unit of force in skinned muscle fibers^34^ may lead to the conclusion that RFE mechanisms counteract the development of fatigue. However, none of the listed studies on voluntary submaximal lengthening contractions lasted for more than 30 s and it is unclear if shown short term effects persist over time. Therefore, we speculated that if stretch-induced reduction of muscle activity can be sustained over an exhausting period of time (>50 s), this increase in neuronal efficiency should have a positive influence on the resistance to the development of peripheral and/or central fatigue. Hence, the aim of this study was to address the question if mechanisms associated with stretch-induced residual force enhancement counteract arising fatigue. We hypothesized active lengthening prior to submaximal exhausting contractions of the human m. quadriceps femoris would lead to reduced muscle activation and reduced fatigability as compared to a pure isometric contraction of identical intensity.

## Methods

### Subjects

Sixteen healthy subjects (7 ♀, 9 ♂; 27 ± 4 years) voluntarily participated in this study and gave written informed consent. None of them had any history of leg and particular knee injury or neurological disorders. The study was approved by the local Ethics Committee of the Technical University of Munich and conducted according to the Declaration of Helsinki.

### Experimental set-up

During all tests, single leg knee extension torque was measured using a motor driven dynamometer (Isomed 2000, D&R Ferstl GmbH, Germany) in isometric or isokinetic mode (60°/s). All subjects were seated upright on the dynamometer with a hip flexion angle of 100° and they were firmly fixed with safety belts. Isometric contractions were performed at 100° knee flexion angle (0° referring to fully extended knee), eccentric contractions were performed over a 20° range of motion, ending at 100°. Bipolar surface electrodes were attached to subjects following the guidelines for preparation and electrode placement of the SENIAM-group^45^. Inter-electrode distance was 2 cm, and EMG data of m. vastus lateralis (VL), m. rectus femoris (RF) and m. vastus medialis (VM) were amplified no further than 10 cm from the recording site (OT bioelettronica, Italy). All data was recorded at a sampling rate of 4 kHz.

### Neuro-mechanical testing

For the assessment of neuromuscular function, voluntary peak torque (MVC) was measured and electrically evoked twitches (femoralis nerve stimulation; 1ms pulse doublets 10ms apart, DS7AH Digitimer constant voltage stimulator, UK) were recorded during the plateau of MVCs (superimposed twitch, SIT) and after deactivation of the muscle in a relaxed state (resting twitch, RT). Electrical stimulus intensity was assessed during twitch-response tests with increasing stimulus current. Supra-maximal stimulus intensity for further tests was set to 150% of the single-pulse stimulus current needed to evoke maximum twitch-torque and maximum VL M-wave peak-to-peak amplitude. The interpolated twitch technique^46^ was used to calculate voluntary activation (VA) as [1 − (SIT / RT)] × 100%.

### Experimental Protocol

All subjects were familiarized with the dynamometer and had to train MVCs and submaximal knee extensions in isometric and isometric-eccentric modes in at least one training session.

**Figure 1:**
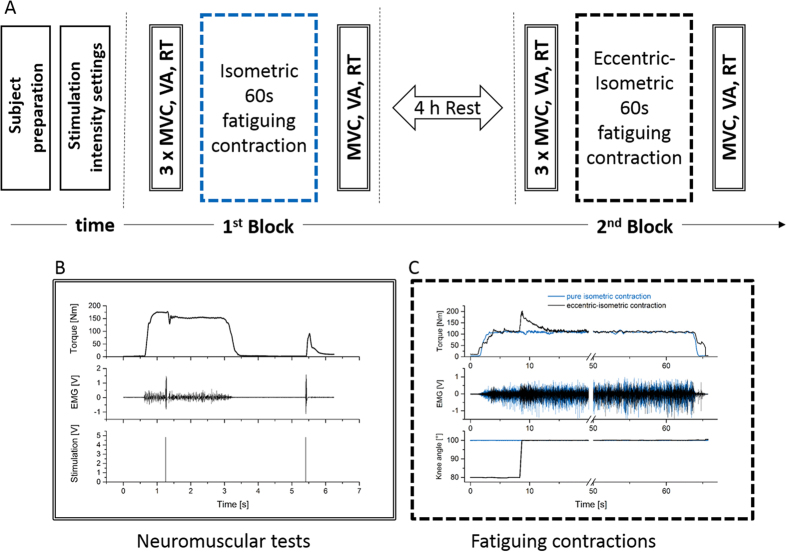
Experimental parts and time line. (**A**) after subject preparation and settings for electrical stimulation, neuromuscular tests (**B**) consisting of maximum voluntary torque (MVC), voluntary activation (VA) and resting twitch torque (RT) were carried out. Thereafter, a 60 s fatiguing contraction (**C**) with (black) and without (blue) preceding lengthening at 60% of MVC was performed in the first and second block, respectively. After fatiguing trials another set of neuromuscular tests was performed. Blocks were separated by four hours rest.

On test day subjects were prepared with EMG electrodes and performed a 10 min general warmup on a bicycle ergometer (100 W), followed by a local warmup on the dynamometer. Thereafter, a set of neuromuscular tests (MVC-ISO-pre) including three MVCs with SITs and RTs were conducted with 3 min rest in between each. After another 5 min rest subjects had to perform a 60 s isometric contraction at an intensity of 60% of previously measured maximum voluntary torque (Fig. 1). During this sub-maximal contraction subjects got visual real-time feedback of their torque output and were asked to match and maintain the given 60% MVC torque level as precisely as possible. Immediately after this fatiguing contraction another neuromuscular test (MVC-ISO-post) was executed in identical manner as MVC-ISO-pre.

Thereafter subjects got four hours of rest in order to fully recover from the first block of experiments. All measurement equipment stayed on subjects, attached to identical positions on the thigh muscle. The second block of experiments also started with a set of three neuromuscular tests (MVC-DYN-pre), identical to MVC-ISO-pre. Subsequently subjects performed a second isometric fatiguing contraction identical to fist block but preceded by a 20° lengthening contraction (60°/s). Once again, fatiguing contraction was immediately followed by a neuromuscular test (MVC-DYN-post).

The order of tests was identical for all subjects and randomization was purposely not undertaken. Although all subjects had a rest of 4 h, we cannot guarantee that all body systems totally recovered. The test design therefore is biased on purpose and a measurable effect of RFE would always have to outperform possible limitations of muscle function due to incomplete recovery.

### Data reduction and analysis

Torque data was smoothed with 20ms moving average and EMG data were bandpass filtered (10-400 Hz, 2^nd^ order Butterworth), rectified and smoothed (250ms moving average). As presented in detail in earlier work^30^, a simplified net-EMG model (i) was used to account for the structural complexity of m. quadriceps femoris. EMG signals of VL, RF and VM were weighted based on literature data on physiological cross-sectional area (PCSA)^47^,^48^ and muscle volume^49^, that is reported to be directly related to maximum muscle force^50^. The used weighting factors in this model are 0.17 for RF, 0.35 for VL and 0.25 for VM. The sum of weighted EMGs is considered as ‘net’ overall activation of the QF (m. vastus intermedius was not taken into account as this part was not measureable via surface EMG).


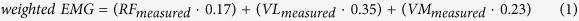


Peak torque of each set of neuromuscular tests was calculated from MVCs and defined as the maximum value before SIT. The set of MVC, SIT and RT of the test with the highest peak torque was used for further statistics. SIT and RT peak torque was derived from the maximum peak-to-base torque difference, with the base defined as mean torque (10ms) at the time point of 10ms before stimulation. The rate of force development in RTs (RFD-RT) was calculated from the maximum slope of torque increase after stimulation. Half-relaxation time (HRT) of evoked RTs was defined as the time from RT peak-torque until torque dropped to50% of peak RT torque.

The pure isometric and eccentric-isometric 60 s endurance trials were synchronized to the beginning of the contraction (50 Nm) and torque, angle, EMG amplitude and EMG median frequency data were then analyzed at five instances in time, every 10 seconds, beginning at the time point corresponding to 10 s after lengthening. EMG data of the 60 s fatiguing trials were normalized to the mean of maximum EMG values measured during the three MVCs preceding respective 60 s trial. Median frequency was assessed using MATLAB codes on power spectral density estimates based on fast Fourier transformations. The mean of five seconds at each time point was used for statistical analysis.

### Statistics

All data was checked for normality (Kolmogorov–Smirnov test) and depending on the outcome either repeated measures ANOVA or nonparametric Friedman tests with post hoc comparisons (Students t-tests or Wilcoxon test) were used to identify significant differences in our data (α ≤ 0.05). Comparisons between neuromuscular tests were assessed between pre vs post fatiguing exercise as well as between pre ISO vs pre DYN, and post ISO vs post DYN. Fatiguing trials were analyzed as within trial comparisons over time, and between trial comparisons concerning contraction conditions: time (5) x condition (2).

## Results

One subject showed incorrect EMG recordings (missing data), and two subjects were not able to keep torque level within 5% of target level throughout the 60 s trial length. These three subjects were discarded from further analysis, resulting in a sample size of n = 13.

### Neuromuscular tests

ANOVA identified statistical differences in MVC, VA, RT, HRT, and RT-RFD when compared over time (before vs after fatiguing trials; p < 0.01), but there were no differences in any of these parameters between conditions before fatiguing trials with and without prior lengthening, and between conditions at time point after fatiguing trials with and without prior lengthening (see Table 1).

### Fatiguing trials

**Figure 2:**
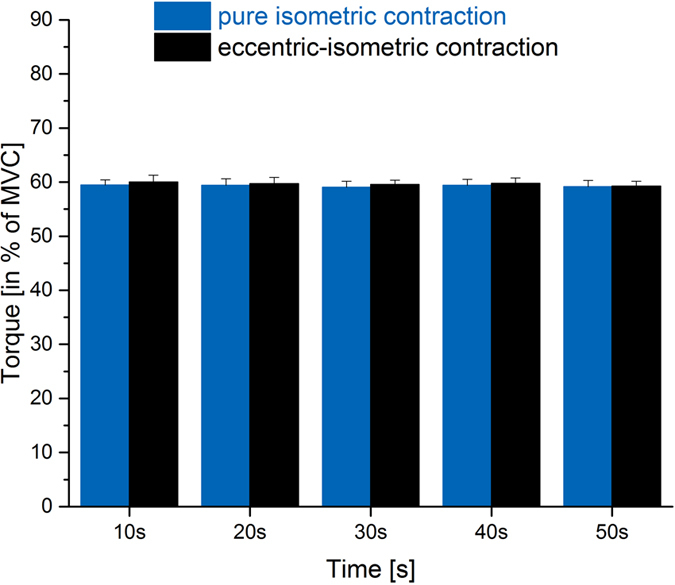
Feedback controlled torque level. Mean and SD of torque (in % of MVC) during 60 s fatiguing trials with (black) and without (blue) prior lengthening. ANOVA identified significant differences in torque control between conditions.

ANOVA statistics identified statistical but negligible differences concerning feedback torque control between conditions (p = 0.015) but not over time (p = 0.113). Mean torque ranged between 59.0 ± 1.1% and 60.0 ± 1.3% of MVC and was slightly higher (<1%) during post-eccentric contractions (Fig. 2).

Median frequency was significantly (p < 0.005) decreasing over time from about 57 to 47 Hz and 64 to 52 Hz for VL and VM, respectively, thereby showing no differences between conditions and no interaction effects. For RF, ANOVA identified a significant decrease (p < 0.005) of median frequency over time from about 56.5 to 40.8 Hz in pure isometric trials, and 56.8 to 43.0 Hz in isometric trials preceded by stretch. There were no interaction effects between time and condition according to RF median frequency, however, a trend (p = 0.067) of difference between conditions with less decrease in RF median frequency after stretch-contraction (Table 2).

Analysis of VL, RF and VM activity revealed a significant increase in activity over time for both fatiguing trials (p < 0.002) and no interaction effects between contraction time and condition. Concerning individual activation reduction in muscle parts, ANOVA identified no differences in conditions for VL (p = 0.259), a trend for VM (p = 0.051), and significantly (p = 0.024) reduced activity of RF after lengthening (Table 2). Paired t-test analysis revealed significantly reduced mean individual activity that reached 83.8 ± 18.7% (p = 0.020), 85.6 ± 14.4% (p = 0.007), 87.9 ± 15.3% (p = 0.017), and 89.6 ± 17.8% (p = 0.047) of the isometric RF reference activation at 10, 20, 30, and 40 s after lengthening, respectively. A non-significant (p = 0.139) RF activation reduction (91.7 ± 22.1% of isometric reference) was found 50 s after lengthening.

**Figure 3:**
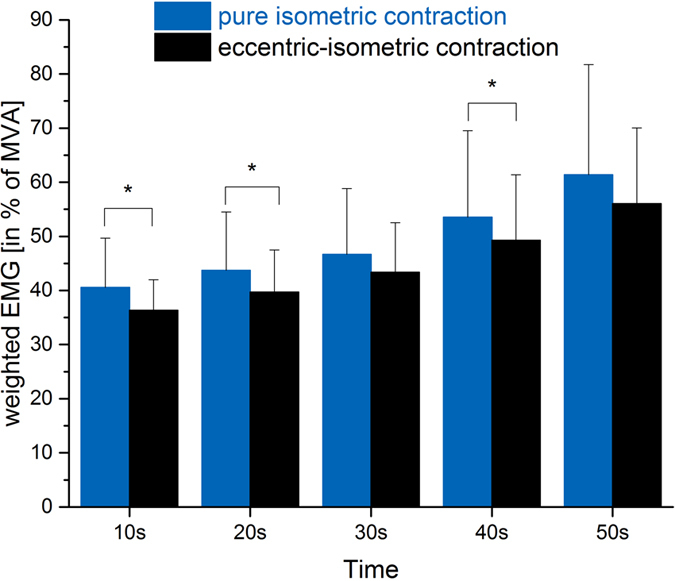
Muscle activation during 60 s fatiguing trials with (black) and without (blue) prior lengthening. Bars represent net quadriceps activations, calculated from weighted and summed EMG data of vastus lateralis, rectus femoris and vastus medialis. ANOVA (post-hoc tests) identified significantly reduced activation after lengthening (*p < 0.05).

Concerning weighted EMG, net activation was significantly (p < 0.036) different between fatiguing trials with and without lengthening, and stretch-induced activation reduction raged between 89 and 92% of pure isometric reference (Fig. 3). Paired t-test analysis identified differences at 10 s (p = 0.018), 20 s (p = 0.019) and 40 s (p = 0.047) after lengthening (Table 2).

## Discussion

The story of residual force enhancement started in the 50’s of the last century and the last word is not spoken yet. Underlying mechanisms remain a matter of debate^2^,^15^,^16^,^51^, and in addition, the relevance of RFE for natural human muscle function is still unclear^35^. Stretch-induced increase in neuromuscular efficiency could be shown in several *in vivo* human studies, indicating optimized muscle function^30^,^31^.

Although mean activation of all measured QF muscle parts was constantly lower after lengthening in this study, only individual contribution of rectus femoris activation reduction was identified by statistical analysis. However, overall net activation of QF in terms of weighted EMG was showing stretch-induced activation reduction for up to 40 s after lengthening (Fig. 3), indicating that the same net torque was achieved with less net neural effort (EMG amplitudes)^30^,^31^,^32^, likely related to reduced metabolic costs^34^.

During fatiguing trials, the contraction intensity was feedback-controlled by subjects at 60% of individual maximum torque. Although there was a statistical difference in torque level between contractions conditions, this difference is virtually of no relevance (Fig. 2). Additionally, it is important to note that there were no differences in peak torque, voluntary activation and potentiated resting twitches in neuromuscular tests before pure isometric and lengthening-isometric fatiguing tasks. This indicates that baseline values and neuromuscular start conditions were identical before fatiguing contractions with and without active lengthening. Furthermore, if there was any uncontrolled influence of insufficient rest between trials, our biased protocol with pure isometric contractions being always the first test would rather lead to an underestimation of stretch-induced activation reduction. Not all measured muscle parts showed statistically significant increases in neuromuscular efficiency, and if so significantly reduced activation was not found for all analyzed time points. This was likely due to the huge standard deviations, indicating a lot of variability in subjects’ muscle activation for solving the task. This limitation may always be part of *in vivo* tests using voluntary muscle activation and needs to be kept in mind when following the discussion of our findings. However, despite obvious variance, the results on net activation are in accordance with previously published work on stretch-induced activation reduction^18^,^30^,^31^,^32^ and add information on *in vivo* muscle function in history dependent scenarios.

It’s highly plausible that the reduced EMG amplitudes found after lengthening trials result from reduced motor unit recruitment and/or lower firing rates during the submaximal muscle action^52^. Accordingly, as force was controlled at a constant level, median frequencies were identical, and net-EMG amplitudes were reduced, a reduced number of active motor units must have produced the controlled force output of 60% MVC. Thus, the force per motor unit and its corresponding muscle fibers was increased. This optimization in neuromuscular efficiency is well in line with the current understanding of which mechanisms might contribute to stretch-induced residual force enhancement. Enhanced force per fiber may partly derive from increased passive stiffness in sarcomeres due to calcium sensitive titin-actin interactions, or be a result of an increased proportion of strongly bound cross-bridges within the contractile machinery^2^,^12^,^15^,^51^,^53^,^54^. Concerning the latter, it seems unlikely that stretch-induced alterations of cross-bridge kinetics persist for 30 seconds and longer, as countless new cross-bridge-cycles take place in post-eccentric phase after lengthening^55^. For this reason and due to the fact that RFE effects are reported to be long-lasting in earlier work^3^,^56^, we favor the idea of a stretch-loaded spring (like titin), that added the most to the force enhancement in active fibers. Consequently, this enhanced force per muscle fiber may have allowed for maintaining a certain load with a reduced number of active motor units and thus net muscle activation. However, this is highly speculative and the performed experiments in this work do not allow for direct conclusions on a microscopic level. Additionally, the used EMG model for net activation of QF muscle needs to be interpreted with caution. Direct evidence of correctness of underlying theoretical base is hardly possible, and although overall results are in accordance with literature, there is no guarantee that our model reflects real muscle activation or coordination. Furthermore, we cannot provide an answer to the questions if, how, and why discussed mechanisms brake up at a certain time, as we did not find reduced activation at the end of our trials. This needs further examination.

Separate from the stretch-induced activation reduction, at all analyzed muscle parts EMG amplitudes increased and median frequencies decreased during the 60 seconds of submaximal muscle action with and without prior lengthening. The continuously decreasing RF, VL and VM median frequencies were not different between contraction conditions at any of the analyzed time points. This indicates that due to earlier fatigue of fast twitch fibers, fiber type recruitment during prolonged muscle action may have changed to a higher proportion slow muscle fibers and decreased muscle fiber conduction velocity over time^57^,^58^. Prolonged half-relaxation times as well as reduced rate of force development in resting twitches after fatiguing trials are well in line with this line of arguments.

Hence, subjects showed typical signs of emerging fatigue over time^59^,^60^,^61^ that resulted in a considerable amount of reduced muscle function. We found a 30% decrease in voluntary maximum torque after 60 s submaximal trials, that derived from reduced voluntary activation levels as well as from severe peripheral fatigue (resting twitches) (Table 1). Interestingly and totally against our expectations, we did not find any difference between neuromuscular tests after the two types of fatiguing contractions. Although neuromuscular efficiency was increased after lengthening, the resulting impairments, especially concerning structural muscle functionality (i.e. resting twitches), were identical after both fatiguing trials. This is in conflict with the idea that stretch-induced enhancement of neuromuscular efficiency counteracts performance fatigability.

To the authors’ knowledge, there is only one study analyzing energetic cost after lengthening-contractions on a muscle fiber level and a decrease in ATPase activity per unit of force was found in isometric contractions following active lengthening^34^. During muscle action ATP is used to maintain Na^+^/K^+^ pumping in and out of a muscle cell after an action potential, to generate force and do work during cross-bridge cycling^62^. It is unclear if comparable mechanisms can be transferred to *in vivo* muscle action and performance fatigability. Concerning our results, it may be speculated that the total intensity and duration of the 60 s fatiguing task at 60% of maximum voluntary torque was too strenuous in both types of contractions, such that a distinction between different states of fatigue was not possible anymore. Indeed, the number of non-responders (i.e. subjects showing no activation reduction at specific time points) increased with increasing contraction time and to the end of the 60 s fatiguing contraction no differences in net activation could be found between conditions. As a result, this may have caused comparable amounts of severe disturbances in the excitation-contraction coupling, depletion of muscle glycogen or accumulation of metabolites, and our tests may not have been sensitive enough to distinguish. However, again, this needs further research.

In conclusion, a stretch-induced increase in neuromuscular efficiency can be supported at least for findings on net activation reduction after active lengthening contractions, although this was not found for all individual muscle parts. After 60 s of muscle action, fatigue signaling parameters were identified irrespective of prior eccentric lengthening and no differences were found between the two types of fatiguing contractions. Hence, no advantage of RFE mechanisms could be identified in terms of better resistance against the development of central or peripheral fatigue. This may be due to the strenuous protocol in this study or huge individual variability. Further work is needed to elaborate if results differ when analyzing additional submaximal contraction intensities in different muscles.

## Additional Information

**How to cite this article**: Seiberl, W. *et al*. Reduced activation in isometric muscle action after lengthening contractions is not accompanied by reduced performance fatigability. *Sci. Rep.* **6**, 39052; doi: 10.1038/srep39052 (2016).

**Publisher's note:** Springer Nature remains neutral with regard to jurisdictional claims in published maps and institutional affiliations.

## Figures and Tables

**Table 1:** Data show neuromuscular tests before and after fatiguing trials with (ECC-ISO) and without (ISO) prior lengthening.

n=13	before ISO		after ISO		before ECC-ISO		after ECC-ISO	
	mean	*SD*	mean	*SD*	mean	*SD*	mean	*SD*
MVC torque [Nm]	139.2	*41.9*	**95.6**	*29.2*	136.6	*40.8*	**95.5**	*26.7*
RT [Nm]	43.1	*22.0*	**29.4**	*16.6*	43.4	*21.9*	**30.3**	*17.3*
VA [%]	94.8	*4.5*	**90.5**	*6.9*	95.9	*4.3*	**90.1**	*9.7*
RFD-RT max [Nm/s]	1029.9	*493.1*	**673.8**	*350.4*	1070.2	*508.8*	**691.4**	*355.7*
HRT [ms]	78.7	*25.8*	**132.7**	*54.1*	82.9	*36.0*	**133.4**	*55.2*

Data shows means and SD of maximum voluntary torque (MVC), resting twitch torque (RT), voluntary activation (VA), rate of force development in RTs (RFD-RT), and half-relaxation times of RTs.

Bold values indicate significant difference to corresponding parameter before fatigue (p<0.01).

**Table 2:** EMG data of 60 s fatiguing trials with (ecc-iso) and without (iso) prior active lengthening.

EMG parameter			10 s		20 s		30 s		40 s		50 s		p value ANOVA	
			mean	*SD*	mean	SD	mean	*SD*	mean	*SD*	mean	*SD*	condition	time
Amplitude [% MVA]	VL	iso	53.4	*9.5*	56.2	*10.6*	59.1	*10.0*	67.0	*12.2*	74.3	*14.6*	0.26	<0.01
		ecc-iso	48.7	*7.9*	52.9	*9.2*	57.3	*9.9*	63.9	*10.8*	70.1	*10.5*		
	RF	iso	60.6	*24.0*	70.0	*26.0*	74.4	*26.3*	84.9	*34.0*	97.8	*45.1*	**0.024**	<0.01
		ecc-iso	**48.4**	*14.4*	**57.7**	*16.8*	**63.5**	*19.3*	**73.8**	*28.3*	85.9	*40.9*		
	VM	iso	50.3	*13.5*	52.8	*15.9*	57.8	*20.8*	68.0	*29.2*	81.6	*40.1*	0.051	<0.01
		ecc-iso	47.5	*9.7*	50.0	*13.9*	55.1	*17.1*	63.6	*24.1*	73.8	*30.9*		
	net-EMG	iso	40.6	*9.1*	43.7	*10.8*	46.6	*12.2*	53.5	*16.0*	61.4	*20.3*	**0.036**	<0.01
		ecc-iso	**36.2**	*5.9*	**39.8**	*8.1*	43.5	*9.5*	**49.5**	*12.6*	56.1	*14.5*		
median frequency [Hz]	VL	iso	56.5	*7.6*	53.7	*7.4*	52.0	*7.6*	50.4	*9.7*	46.7	*8.7*	0.29	<0.01
		ecc-iso	57.2	*6.9*	54.3	*5.7*	53.4	*6.3*	51.3	*6.7*	48.2	*7.1*		
	RF	iso	56.5	*5.8*	52.3	*6.9*	50.0	*6.3*	45.2	*6.8*	40.8	*6.2*	0.067	<0.01
		ecc-iso	56.8	*6.3*	53.1	*6.6*	50.9	*6.1*	46.1	*7.8*	43.0	*6.0*		
	VM	iso	63.7	*13.7*	61.6	*12.6*	59.7	*11.8*	56.0	*11.7*	51.9	*11.5*	0.95	<0.01
		ecc-iso	64.7	*13.1*	61.1	*11.6*	58.9	*11.1*	55.7	*11.3*	52.2	*12.2*		

Data shows mean and SD of EMG amplitude and frequeny parameters of vastus lateralis (VL), rectus femoris (RF) and vastus medialis (VM).

Bold values indicate significant difference to isometric references (p < 0.05).
